# Hypertrophic cardiomyopathy is not the sole echocardiographic phenotype associated with hyperthyroidism in cats: a retrospective study in 147 cats (2005-2025)

**DOI:** 10.1093/jvimsj/aalag006

**Published:** 2026-02-16

**Authors:** Pierre Foulex, Benjamin Reslinger, Maxime Kurtz, Emilie Trehiou, Camille Poissonnier, Peggy Passavin, Kahina Kartout, Sarra Ghazal, Christelle Maurey, Ghita Benchekroun, Thibault Ribas, Loïc Desquilbet, Valérie Chetboul

**Affiliations:** Veranex France, Paris, France; Clinique vétérinaire Boulogne Roland-Garros, Boulogne-Billancourt, France; Sudvetia, Aix-en-Provence, France; Ecole Nationale Vétérinaire d’Alfort, CHUV-AC, Maisons-Alfort, France; Ecole Nationale Vétérinaire d’Alfort, CHUV-AC, Maisons-Alfort, France; Ecole Nationale Vétérinaire d’Alfort, CHUV-AC, Maisons-Alfort, France; Ecole Nationale Vétérinaire d’Alfort, CHUV-AC, Maisons-Alfort, France; Clinique vétérinaire Boulogne Roland-Garros, Boulogne-Billancourt, France; Ecole Nationale Vétérinaire d’Alfort, CHUV-AC, Maisons-Alfort, France; Ecole Nationale Vétérinaire d’Alfort, CHUV-AC, Maisons-Alfort, France; Ecole Nationale Vétérinaire d’Alfort, CHUV-AC, Maisons-Alfort, France; Ecole Nationale Vétérinaire d’Alfort, CHUV-AC, Maisons-Alfort, France; Sudvetia, Aix-en-Provence, France; Ecole Nationale Vétérinaire d’Alfort, Univ Paris Est Créteil, INSERM, IMRB, Maisons-Alfort, France; Ecole Nationale Vétérinaire d’Alfort, CHUV-AC, Maisons-Alfort, France; Ecole Nationale Vétérinaire d’Alfort, Univ Paris Est Créteil, INSERM, IMRB, Maisons-Alfort, France

**Keywords:** congestive heart failure, echocardiography, heart, myocardial disease, restrictive cardiomyopathy, thyroxine

## Abstract

**Background:**

A hypertrophic cardiomyopathy phenotype (HCMP) can occur in cats with hyperthyroidism. However, it remains unclear whether other cardiomyopathy phenotypes are also associated with hyperthyroidism in cats.

**Hypothesis/Objectives:**

Describe the epidemiological, clinical, and echocardiographic findings and cardiomyopathy phenotypes in a large sample of hyperthyroid cats. Compare the echocardiographic features of hyperthyroid cats with HCMP to those of a contemporaneous sample of normotensive euthyroid cats with primary hypertrophic cardiomyopathy (HCM).

**Animals:**

A total of 147 hyperthyroid cats and 112 cats with primary HCM.

**Methods:**

Retrospective study with review of internal medicine and cardiology service databases (2005-2025).

**Results:**

Most hyperthyroid cats (117/147, 80%) exhibited 1 of the 3 cardiomyopathy phenotypes: HCMP (94/147, 64%), restrictive cardiomyopathy phenotype (RCMP; 20/147, 14%), or nonspecific (3/147, 2%). Hyperthyroid cats with RCMP had significantly higher total thyroxine concentrations (median, 154 nmol/L vs 95 nmol/L) and more dyspnea related to congestive heart failure (80% vs 11%) than those with HCMP (*P* < .01). A gallop sound was detected in 10% of hyperthyroid cats (14/147), exclusively in those with HCMP (10%, 9/94) or RCMP (25%, 5/20). The end-diastolic left ventricular diameter was higher in hyperthyroid cats with HCMP than in those with primary HCM (*P* < .01). Subaortic septal hypertrophy was more frequent (95% vs 67%) in cats with primary HCM than in those with hyperthyroidism-associated HCMP (*P* < .01).

**Conclusions and clinical importance:**

The HCMP is the predominant, but not sole, echocardiographic phenotype observed in hyperthyroid cats. The RCMP is the second most frequent phenotype and may reflect a more severe form of hyperthyroidism.

## Introduction

Hyperthyroidism is the most commonly diagnosed endocrinopathy in geriatric cats and is frequently associated with cardiac abnormalities, potentially leading to congestive heart failure (CHF).^1,2^ One study including 91 hyperthyroid cats determined that echocardiographic abnormalities before treatment included increased end-diastolic interventricular septum (IVS), left ventricular free wall (LVFW) thicknesses or both, a pattern also defined as a hypertrophic cardiomyopathy phenotype (HCMP), with reversion toward normal values after radioiodine treatment.^3^ In addition to hyperthyroidism-induced HCMP, the restrictive cardiomyopathy phenotype (RCMP) has been proposed as a potential cardiac manifestation in cats with hyperthyroidism by the American College of Veterinary Internal Medicine (ACVIM) consensus statement panel on cardiomyopathy in cats.^4^ However, to our knowledge, no study has yet investigated this assertion.

The objectives of our retrospective study were therefore: (1) to describe the epidemiological, clinical, and echocardiographic findings, as well as the cardiomyopathy phenotypes (CMPs) associated with hyperthyroidism in cats, and (2) to compare the echocardiographic features of hyperthyroidism-associated HCMP cats to those of a contemporaneous sample of normotensive euthyroid cats with primary hypertrophic cardiomyopathy (HCM), similar in terms of age and body weight (BW).

## Materials and methods

### Study design

Ours was a retrospective, multicenter study. Electronic medical records from 3 referral cardiology centers were reviewed for cats diagnosed with hyperthyroidism between 2005 and 2025.

### Case selection and review

Cats were retrospectively included if they met both inclusion criteria: (1) a diagnosis of hyperthyroidism confirmed by a board-certified specialist in internal medicine (European College of Veterinary Internal Medicine-Companion Animals [ECVIM-CA] diplomate—internal medicine) based on serum total thyroxine (T4) concentration above the upper limit of the laboratory reference interval and clinical signs consistent with hyperthyroidism; and (2) a standard transthoracic echocardiogram (M-mode, 2-dimensional [2D], and conventional Doppler examination) performed or supervised by a board-certified specialist in cardiology (ECVIM-CA diplomate—cardiology) in a referral cardiology department.

Cats were not included in the study if the date of the echocardiogram or T4 measurement was unknown, if the echocardiogram was performed > 45 days before the diagnosis of hyperthyroidism, or if they had been treated for hyperthyroidism for > 45 days before the echocardiographic examination. A complete medical report was mandatory (history, patient birth date, weight, breed, clinical examination findings, T4 measurement, and standard transthoracic echocardiographic examination), and cats were not included in the study if cardiac auscultation was not documented in the cardiology department.

The time between echocardiographic examination, serum T4 measurement, and initiation of antithyroid treatment was mandatory and systematically recorded.

Echocardiography was performed by trained operators in unsedated, minimally handled cats positioned comfortably in a standing position as previously described and recommended by the ACVIM consensus statement on cardiomyopathies in cats.^4,5^ Data on age, sex, BW, and clinical findings were recorded. In both participating centers, all measurements were systematically reviewed and validated by a board-certified cardiologist, ensuring consistency and reliability of the data.

Our retrospective study was based on inclusion of all eligible cases over a 20-year period (medical record review beginning in 2005, when electronic reports became available). The sample size therefore was dictated solely by the number of cats fulfilling the predefined inclusion and exclusion criteria.

### Echocardiographic assessment

A standard echocardiographic examination (2D, M-mode, and Doppler examination) was performed in all cats by a board-certified veterinary cardiologist, a cardiology resident under direct supervision of a board-certified cardiologist, or a trained observer with at least 6 years of experience in echocardiography, using different ultrasonographic units (Vivid 7, Vivid E9, Vivid iQ, and Vivid E95, General Electric Medical System, Waukesha, WI, USA).

M-mode-derived left ventricular (LV) linear dimensions and fractional shortening,^4^ 2D end-diastolic subaortic IVS thickness,^6,7^ end-diastolic left atrium-to-aorta ratio (LA:Ao) and right atrial diameter,^5–9^ and Doppler-derived early (E) and late (A) mitral flow velocities and E:A ratio^10^ were obtained in unsedated cats in standing position. Left atrial (LA) enlargement was defined as end-diastolic LA:Ao > 1.2^7^ and right atrial enlargement was diagnosed if the right atrial diameter measured at end-diastole at the level of the tricuspid annulus exceeded 15 mm.^8,9^ Dynamic LV outflow tract obstruction was defined as an aortic velocity > 2 m/s.^7^ Color-flow and spectral Doppler modes also were used to identify mitral or tricuspid valve regurgitation, as well as shunting and stenotic lesions.

### Cardiomyopathy phenotypes

According to the ACVIM consensus statement guidelines on cardiomyopathies in cats,^4^ HCMP was defined as diffuse or regional increased LV wall thickness with a nondilated LV chamber.^4^ To analyze myocardial thicknesses and LV diameters, M-mode parameters of each cat were compared to the 95% prediction intervals according to BW established from a large cohort of healthy cats.^11^ In addition, subaortic IVS hypertrophy was diagnosed when the absolute value of 2D end-diastolic subaortic IVS thickness was superior to the 95% prediction intervals of end-diastolic IVS according to BW established in the aforementioned study. Symmetrical LV hypertrophy was defined as an end-diastolic IVS-to-LVFW ratio between 0.7 and 1.3.^12^

As stated in the ACVIM consensus statement guidelines on cardiomyopathies in cats, the myocardial form of RCMP was defined by normal LV dimensions (including wall thickness) with LA or biatrial enlargement.^4^ The dilated CMP was defined as LV systolic dysfunction characterized by an end-systolic LV internal diameter above the 95% prediction intervals according to BW established from a large cohort of healthy cats, with decreased fractional shortening (ie, < 28%).^4,11^ The arrhythmogenic CMP was characterized by right atrial and ventricular dilatation with right ventricular systolic dysfunction and wall thinning.^4^ Lastly, a CMP not adequately described by the previous categories was termed the nonspecific CMP (NSCMP), as recommended.^4^ Cats with no CMP were assigned to the No CMP group.

### Systemic arterial blood pressure

Systolic systemic arterial blood pressure (SBP) was noninvasively assessed by Doppler method (811-BL, Parks Medical Electronics, Inc., Aloha, Oregon, USA) and the mean SBP value was used for statistical analysis.^13^ Systemic hypertension was classified as moderate when SBP ranged from 160 to 179 mmHg, and as severe when SBP was equal to or exceeded 180 mmHg.^13^

### Contemporary sample of normotensive euthyroid cats with suspected primary HCM

To compare echocardiographic features between hyperthyroidism-associated HCMP and primary HCM, a contemporary sample of normotensive euthyroid cats with primary HCM was retrospectively enrolled.

To ensure comparability, these cats had to be at least 9 years old, weigh ≤ 6 kg, and be contemporaneous with the main study sample. They had to be diagnosed with an HCM phenotype using the same criteria as described above for the main study sample. To be ultimately included in the primary HCM group, cats were required to have a T4 concentration within the reference interval, normal SBP, and no evidence of any other systemic illness. These tests had to be performed no more than 1 month before or after the echocardiographic diagnosis of HCM.

### Statistical analysis

Statistical analyses were performed using RStudio software. Categorical data are reported as proportions or percentages, whereas continuous data are presented as medians with IQRs. Associations between CMP (categorized into 3 groups: HCMP, RCMP, and No CMP) and epidemiological, clinical, or echocardiographic variables were assessed using either the chi-squared or Fisher’s exact test for categorical variables, and the Kruskal–Wallis test for continuous variables. The association between CMP and heart murmur grade was analyzed using the ordinal Kruskal–Wallis test. Post-hoc pairwise comparisons (using the chi-squared or Fisher’s exact tests for categorical variables, and the Mann–Whitney test for continuous variables) were performed only when a significant overall association was identified, in order to control the type I error rate.^14,15^ In addition, to control for multiple comparisons, hypothesis testing was not performed for all variables but only for those that directly addressed the study objectives or for which a priori hypotheses had been formulated.

Correlation between T4 and echocardiographic variables was assessed using Spearman correlation analysis. The prevalences of CHF signs and cardiac-related thromboembolic events (ie, those associated with LA enlargement) were compared only between the HCMP and RCMP groups, based on the a priori assumption that their prevalence in the No CMP group should be zero.

Comparisons between cats with hyperthyroidism-associated HCMP and those with primary HCM were conducted using either the chi-squared or Fisher’s exact tests for categorical variables, and the Mann–Whitney test for continuous variables. When differences between groups reached statistical significance, echocardiographic variables were normalized to both BW and age, in order to assess the association between the echocardiographic variable and the group independently of age and BW.

The alpha type-I error was set at 0.05.

## Results

### Study sample of hyperthyroid cats

**Figure 1:**
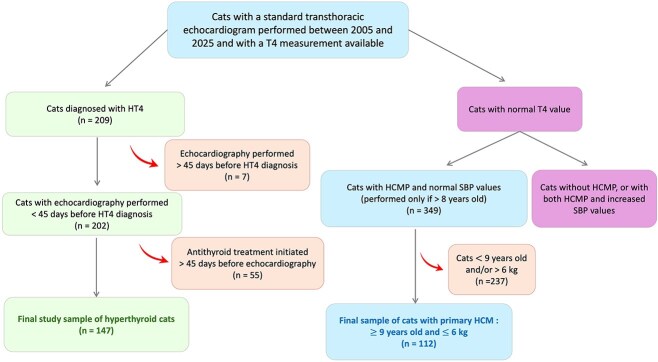
Flowchart illustrating the recruitment of cats included in the study. The medical record searching was performed as follows: cats undergoing a standard transthoracic echocardiographic examination between 2005 and 2025 with a total thyroxine (T4) measurement available were screened for eligibility. Hyperthyroid cats (HT4) were included if echocardiography was performed within 45 days before the diagnosis of hyperthyroidism and if no antithyroid treatment had been initiated more than 45 days before the examination. Cats with normal T4 concentrations were included in the primary HCM group if they presented with HCMP, had normal systolic SBP values (performed if > 8 years old), were ≥ 9 years of age, and weighed ≤ 6 kg. Cats not meeting these clinical, echocardiographic, or demographic criteria were excluded from the study. Abbreviations: HCM = hypertrophic cardiomyopathy; HCMP = hypertrophic cardiomyopathy phenotype; SBP = systemic blood pressure.

A total of 229 cats diagnosed with hyperthyroidism and with an echocardiography report available were identified for potential inclusion during the study period (2005-2025). Of these, 20 were excluded because the T4 concentration at diagnosis was not available, 7 because echocardiography was performed > 45 days before the hyperthyroidism diagnosis, and 55 because antithyroid treatment was initiated for > 45 days before echocardiography (Figure 1).

Finally, the study sample of hyperthyroid cats (Table 1) consisted of 147 cats (median age [IQR] = 14.2 years [12.7-15.8], BW = 3.7 kg [2.9-4.7], female = 50% (73/147), and T4 = 98 nmol/L [74-150]). Most cats were non-pedigree (131/147, 89%), with the Siamese being the most common pedigree breed (5/147, 3%).

**Table 1:** Epidemiological and clinical features of hyperthyroid cats according to their cardiomyopathy phenotypes as defined by the American College of Veterinary Internal Medicine consensus statement on feline cardiomyopathies.^4^

**Characteristic**		**NSCMP** **2%** **(*n* = 3)**	**Entire population** **100%** **(*n* = 147)**	**HCMP** **64%** **(*n* = 94)**	**RCMP** **14%** **(*n* = 20)**	**No CMP** **20%** **(*n* = 30)**	***P*** ^**b**^
**Sex % (number)**	*Female*	100% (3/3)	50% (73/147)	48% (45/94)	35% (7/20)	60% (18/30)	.22
**Age (years)**		14.8 [14.7-15.2]	14.2 [12.7-15.8]	14.3 [12.9-16.0]	14.5 [12.4-16.1]	13.9 [11.9-15.5]	.53
**Body weight (kg)**		4.5 [4.1-4.6]	3.7 [2.9-4.7]	4.0 [2.7-4.8]	3.4 [3.1-4.3]	4.0 [3.1-4.6]	.76
**Time between echocardiography and hyperthyroidism diagnosis (days)**		5 [−2-7]	−3 [−14-0]	−3 [−14-0]	0 [−6.8-0]	−10 [−25-(−1)]	^c^
**Time between antithyroid therapy and echocardiography (days)**		−5 [−7-2]	1 [0-13]	1 [0-12]	0 [0-6.8]	4 [0-22]	^c^
**Total thyroxine level (nmol/L)**		94 [75-202]	98 [74-150]	95 [74-134]	154 [88-218]	93 [72-132]	< .01
**Presence of gallop sound % (number)**		0% (0/3)	10% (14/147)	10% (9/94)	25% (5/20)	0% (0/30)	^c^
**Presence of a systolic heart murmur % (number)**		67% (2/3)	84% (124/147)	85% (80/94)	80% (16/20)	87% (26/30)	^c^
**Heart murmur grade (1-6)**		2 (1-2)	3 (2-4)	4 (2-4)	2 (2-3)	3 (2-3)	<.01
**Systolic heart murmur** ^**a**^ **localization % (number)**	*Left apical*	50% (1/2)	65% (80/124)	65% (52/80)	75% (12/16)	58% (15/26)	^c^
	*Left midthoracic*	0% (0/2)	4% (5/124)	5% (4/80)	0% (0/16)	4% (1/26)	^c^
	*Sternal*	50% (1/2)	29% (36/124)	30% (22/80)	19% (3/16)	38% (10/26)	^c^
	*Right apical*	0% (0/2)	4% (5/124)	5% (4/80)	6% (1/16)	0% (0/26)	^c^
**Congestive heart failure % (number)**	*Pulmonary edema*	0% (0/3)	9% (13/147)	5% (5/94)	40% (8/20)	0% (0/30)	<.01^d^
	*Pleural effusion*	33% (1/3)	11% (16/147)	5% (5/94)	50% (10/20)	0% (0/30)	<.01^d^
	*Pericardial effusion*	0% (0/3)	5% (8/147)	4% (4/94)	20% (4/20)	0% (0/30)	.03^d^
**Respiratory signs % (number)**	*Dyspnea*	33% (1/3)	18% (27/147)	11% (10/94)	80% (16/20)	0% (0/30)	<.01^d^
	*Paradoxical breathing*	33% (1/3)	8% (12/147)	5% (5/94)	30% (6/20)	0% (0/30)	<.01^d^
**Intracardiac thrombus or arterial thromboembolism % (number)**		0% (0/3)	3% (4/147)	1% (1/94)	15% (3/20)	0% (0/30)	.02^d^
**Systolic systemic arterial blood pressure (mmHg)**		/ /	190 [162-220]	190 [162-220]	200 [160-240]	190 [165-220]	.86

^a^Two cats presented with 2 distinct heart murmurs. ^b^The associations between cardiomyopathy phenotypes and clinical/echocardiographic variables were only tested between HCMP, RCMP, and No CMP groups (due to the low number of cats in the NSCMP group, ie, *n* = 3) and the corresponding *P* value is displayed in the last column. ^c^These associations were not tested either because evaluating them was not an objective of the study and no hypotheses were formulated a priori or because the number of cats in a subgroup was < 5. ^d^The prevalences of congestive heart failure signs and cardiac-related thromboembolic events were compared only between the HCMP and RCMP groups (with corresponding *P*-values shown), based on the a priori hypothesis that their prevalence in the No CMP group should be zero.

Categorical data are presented as percentage (ratio) and continuous data as median [IQR].

Abbreviations: HCMP = hypertrophic cardiomyopathy phenotype; RCMP = restrictive cardiomyopathy phenotype; NSCMP = nonspecific cardiomyopathy phenotype; No CMP = absence of cardiomyopathy phenotype.

The median time between echocardiography and hyperthyroidism diagnosis was −3 days [−14 to 0], and the median time between initiation of antithyroid treatment and echocardiography was 1 day [0-13].

A systolic heart murmur was detected in 84% (124/147) and a gallop sound in 10% (14/147) of the hyperthyroid cats at the time of echocardiography (Table 1). Regarding heart murmur characteristics, the median grade was 3/6 [2-4], and the most common type was a left apical systolic heart murmur (65%, 80/124).

### Echocardiographic features and cardiomyopathy phenotypes in the hyperthyroid study sample

Echocardiography determined that most hyperthyroid cats (117/147, 80%) exhibited 1 of the 3 CMP: HCMP (94/147, 64%), RCMP (20/147, 14%), or NSCMP (3/147, 2%). The remaining 20% (30/147) showed no myocardial abnormalities (No CMP group).

Epidemiological and clinical variables according to CMP are presented in Table 1. In addition, the main echocardiographic and clinical features according to ACVIM stages are summarized in Table 2 for the 2 most common CMP (ie, HCMP and RCMP).

**Table 2:** Epidemiological, clinical and echocardiographic phenotypes of the hyperthyroid cats with HCMP and RCMP subclassified into ACVIM stages.^4^

**Characteristic**		**Cats with HCMP** **64%** **(*n* = 94)**				**Cats with RCMP** **14%** **(*n* = 20)**			***P*** ^**a**^
		**All HCMP cats** **(*n* = 94)**	**ACVIM** **stage B1 (*n* = 68)**	**ACVIM** **stage B2** **(*n* = 16)**	**ACVIM** **stage C** **(*n* = 10)**	**All RCMP cats** **(*n* = 20)**	**ACVIM** **stage B2** **(*n* = 4)**	**ACVIM** **stage C** **(*n* = 16)**	
**Sex**	*Female*	48% (45/94)	56% (38/68)	19% (3/16)	40% (4/10)	35% (7/20)	25% (1/4)	38% (6/16)	^**b**^
**Age (years)**		14.3 [12.9-16.0]	14.3 [13.1-16.4]	14.0 [11.9-15.2]	14.0 [10.4-16.8]	14.5 [12.4-16.1]	14.1 [13.4-14.4]	15.3 [12.4-16.4]	^**b**^
**Body weight (kg)**		4.0 [2.7-4.8]	3.7 [2.7-4.6]	4.0 [3.0-4.8]	4.6 [3.6-5.3]	3.4 [3.1-4.3]	3.4 [3.1-3.7]	3.4 [3.1-4.7]	^**b**^
**Total thyroxine level (nmol/L)**		95 [74-134]	86 [66-117]	107 [90-157]	130 [98-178]	154 [88-218]	98 [62-136]	192 [105-232]	<.01
**Congestive heart failure % (number)**	*Pulmonary edema*	5% (5/94)	0% (0/68)	0% (0/16)	50% (5/10)	40% (8/20)	0% (0/4)	50% (8/16) 63% (10/16)	<.01
	*Pleural effusion*	5% (5/94)	0% (0/68)	0% (0/16)	50% (5/10)	50% (10/20)	0% (0/4)		<.01
	*Pericardial effusion*	4% (4/94)	0% (0/68)	0% (0/16)	40% (4/10)	20% (4/20)	0% (0/4)	25% (4/16)	.03
**Intracardiac thrombus or arterial thromboembolism % (number)**		1% (1/94)	0% (0/68)	0% (0/16)	10% (1/10)	15% (3/20)	0% (0/4)	19% (3/16)	.02
**Respiratory signs % (number)**	*Dyspnea*	11% (10/94)	0% (0/68)	0% (0/16)	100% (10/10)	80% (16/20)	0% (0/4)	100% (16/16)	<.01
	*Paradoxical breathing*	5% (5/94)	0% (0/68)	0% (0/16)	50% (5/10)	30% (6/20)	0% (0/4)	38% (6/16)	<.01
**M-mode echocardiographic variables**	*LVIDd (mm)*	14.7 [13.3-16.3]	14.1 [13.1-16.0]	17.0 [15.4-18.6]	14.7 [13.6-16.0]	17.0 [15.4-18.6]	16.5 [14.6-17.7]	17.0 [15.4-19.2]	<.01
	*IVSd (mm)*	5.8 [4.9-6.3]	5.7 [4.9-6.2]	5.9 [4.7-6.1]	5.9 [5.4-6.7]	4.2 [4.0-4.5]	3.9 [3.6-4.3]	4.3 [4.1-4.5]	<.01
	*LVFWd (mm)*	5.4 [4.8-6.0]	5.2 [4.6-5.7]	5.7 [5.4-6.0]	7.2 [6.7-7.8]	4.5 [4.1-4.8]	4.1 [3.7-4.2]	4.5 [4.3-4.9]	<.01
	*FS (%)*	53 [45-59]	53 [46-59]	53 [45-60]	54 [46-59]	47 [41-59]	46 [45-50]	48 [41-59]	^**b**^
**Two-dimensional echocardiographic variables**	*End-diastolic LA:Ao ratio*	1.0 [0.9-1.2]	1.0 [0.9-1.1]	1.4 [1.3-1.6]	1.9 [1.5-2.1]	1.7 [1.5-2.1]	1.5 [1.4-1.5]	2.0 [1.6-2.2]	<.01
	*End-diastolic RA diameter (mm)*	10.4 [9.1-11.7]	10.0 [8.7-11.0]	10.5 [10.1-13.5]	14.3 [12.0-15.4]	14.8 [14.6-15.9]	14.4 [13.0-15.7]	14.8 [14.6-15.9]	<.01
	*SA-IVSd (mm)*	5.6 [4.9-6.6]	5.6 [4.9-6.3]	5.6 [5.1-6.8]	5.6 [4.7-7.2]	4.5 [4.1-4.9]	4.7 [4.4-5.0]	4.5 [4.1-4.9]	<.01
**Conventional Doppler variables**	*Peak aortic flow velocity (m/s)*	1.4 [1.1-2.1]	1.4 [1.1-2.0]	1.9 [1.4-3.1]	1.2 [1.0-1.4]	1.3 [0.9-1.4]	1.3 [1.3-1.3]	1.4 [0.9-1.5]	.07
	*Mitral E:A ratio* ^c^	0.8 [0.7-1.1]	0.7 [0.6-1.0]	0.8 [0.8-1.4]	0.9 [0.8-0.9]	2.9 [2.2-3.2]	/ /	2.9 [2.2-3.2]	<.01

^a^For each variable, the comparison between all HCMP cats and all RCMP cats was only tested when the overall association between the variable and the 3 most common echocardiographic groups (ie, HCMP, RCMP, and absence of cardiomyopathy phenotype [No CMP] groups) reached significance, and the corresponding *P* value is displayed in the last column. ^b^The comparison was not tested because the overall association between the variable and the CMP group did not reach significance. ^c^44/94 HCMP cats and 7/20 RCMP cats exhibited unfused mitral E and A waves, allowing calculation of the mitral E:A ratio in these individuals.

Abbreviations: ACVIM = American College of Veterinary Internal Medicine; FS = fractional shortening; HCMP = hypertrophic cardiomyopathy phenotype; IVSd = end-diastolic interventricular septum thickness; LA:Ao ratio = left atrium-to-aorta ratio; LVFWd = end-diastolic left ventricular free wall thickness; LVIDd = end-diastolic left ventricular internal diameter; Mitral E:A ratio = early (E)-to-late (A) diastolic mitral inflow velocities ratio; RA = right atrial; RCMP = restrictive cardiomyopathy phenotype; SA-IVSd = end-diastolic subaortic interventricular septum thickness.

No significant differences were found among the 3 most common echocardiographic groups (HCMP, RCMP, and No CMP groups) regarding sex, age, and BW (Table 1). The median heart murmur grade (Table 1) differed significantly (*P* < .01) among the HCMP (median = 4 [2-4]), RCMP (median = 2 [2-3]), and No CMP groups (median = 3 [2-3]).

A gallop sound was detected exclusively in hyperthyroid cats with HCMP (10%) and RCMP (25%), and was absent in both NSCMP and No CMP cats.

The median T4 was significantly associated with the CMP (*P < .*01). The median T4 was higher (*P* < .01) in the RCMP group (154 nmol/L [88-218]) than in the HCMP group (95 nmol/L [74-134]), and higher (*P = .*01) in the RCMP group than in the No CMP group (93 nmol/L [72-132]). In the entire study sample of hyperthyroid cats, a weak positive correlation was found between T4 and end-diastolic LV diameter (*r_s_* = 0.29; *P* < .01). A moderate positive correlation also was observed between T4 and LA (*r_s_* = 0.45; *P* < .01), LA:Ao (*r_s_* = 0.36; *P* < .01), and E wave velocity (*r_s_* = 0.56; *P* < .01).

Among hyperthyroid cats with available SBP data (*n* = 113), 90 (80%) had SBP $\ge$ 160 mmHg, and the majority of these (72/90, 80%) have SBP $\ge$ 180 mmHg.

No significant difference was found in the prevalence of systemic arterial hypertension (SBP $\ge$ 160 mmHg) among the HCMP (79%, 60/76), RCMP (77%, 10/13), and No CMP groups (82%, 18/22; *P* = 1.00). Similarly, no significant difference was found in the prevalence, respectively, of moderate (SBP between 160 and 179 mmHg; *P* = .92) and severe (SBP $\ge$ 180 mmHg; *P* = 1.00) systemic hypertension among the HCMP group (16%, 12/76 and 63%, 48/76, respectively), the RCMP group (15%, 2/13 and 62%, 8/13, respectively), and the No CMP group (18%, 4/22 and 64%, 14/22, respectively). In addition, the median SBP did not differ significantly (*P* = .86) among the HCMP group (190 mmHg [162-220]), the RCMP group (200 mmHg [160-240]), and the No CMP group (190 mmHg [165-220]; Table 1).

Dyspnea (80% vs 11%; *P* < .01) and paradoxical breathing (30% vs 5%; *P* < .01), both related to CHF, were significantly more frequent in RCMP cats than in HCMP cats (Table 1), with pulmonary edema observed in 40% vs 5% (*P* < .01), pleural effusion in 50% vs 5% (*P* < .01), and pericardial effusion in 20% vs 4% (*P* = .03). Similarly, the occurrence of intracardiac thrombus or arterial thromboembolism was significantly more frequent in RCMP cats (15%) than in HCMP cats (1%; *P* = .02).

**Figure 2:**
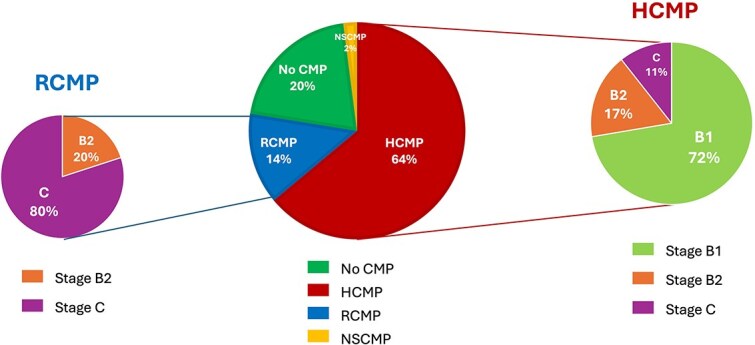
Distribution of cardiomyopathy phenotypes and ACVIM stages of the study sample (*n* = 147 hyperthyroid cats).^4^ Abbreviations: HCMP = hypertrophic cardiomyopathy phenotype; No CMP = no cardiomyopathy phenotype; NSCMP = nonspecific cardiomyopathy phenotype; RCMP = restrictive cardiomyopathy phenotype.

According to the ACVIM classification (Table 2; Figure 2), most HCMP cats (72%, 68/94) were classified as stage B1, whereas 17% (16/94) and 11% (10/94) were in stage B2 and decompensated stage C, respectively.

Median T4 concentration differed significantly among HCMP ACVIM stages (*P* < .01), increasing from stage B1 (86 nmol/L [66-117]) to stage B2 (107 nmol/L [90-157] and stage C (130 nmol/L [98-178]).

The majority of HCMP cats (73%, 69/94) had symmetrical hypertrophy, whereas 15% (14/94) had predominantly the IVS hypertrophy, 4% (4/94) had predominantly the LVFW hypertrophy, and 7% (7/94) showed focal subaortic IVS hypertrophy only. In the HCMP group, the median peak aortic flow velocity was 1.4 m/s [1.1-2.1], and dynamic LV outflow tract obstruction was identified in 26% (24/94).

**Figure 3:**
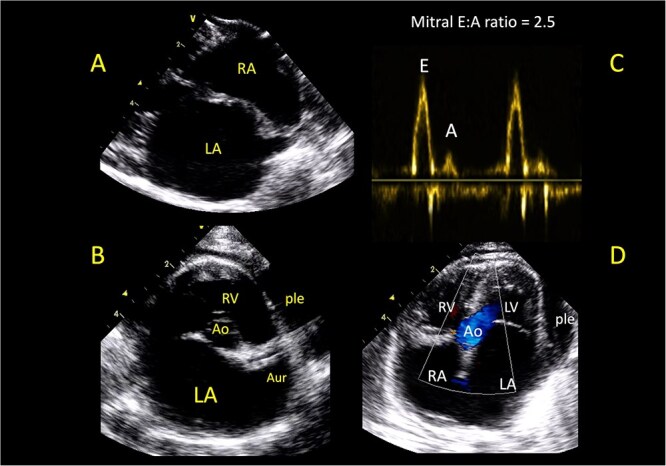
Two-dimensional (2D) echocardiographic right parasternal long-axis 4-chamber view (A), short-axis view optimized for the aortic valve and left atrium (B), transmitral pulsed-wave Doppler examination (C), and 2D left apical 5-chamber view at end-systole (D) obtained in a cat with a hyperthyroidism-associated restrictive cardiomyopathy phenotype. The echo-Doppler examination shows marked biatrial enlargement (A, B, D), a typical restrictive filling pattern (C) of the transmitral flow (E:A ratio = 2.5), and the presence of pleural effusion (ple, B, D), consistent with congestive heart failure. Abbreviations: Ao = aorta; Aur = left auricle; LA = left atrium; LV = left ventricle; RA = right atrium; RV = right ventricle.

Among the RCMP cats, 80% (16/20) presented with acute or chronic CHF and were classified as ACVIM stage C, whereas 20% (4/20) were diagnosed at the preclinical ACVIM stage B2 (Figure 2). All RCMP cats had LA dilatation (end-diastolic LA:Ao = 1.7 [1.5-2.1]). Right atrial diameter was measured in 90% (18/20) of the RCMP cats, and right atrial dilatation was observed in 33% (6/18) of them (Figure 3). Within the RCMP group, the median mitral E:A ratio and fractional shortening were 2.9 [2.2-3.2] and 47% [41-59], respectively.

Three cats exhibited a CMP that did not fit any of the 4 defined categories and therefore were classified as having a NSCMP. One cat had focal subaortic IVS hypertrophy, dilated end-diastolic LV diameter, normal peak aortic flow velocity and LA:Ao ratio, and fractional shortening at the lower limit of the reference interval (28%).^11^

The 2 other cats had LA dilatation with trace mitral valve regurgitation, normal LV diameters and thicknesses, normal fractional shortening, and right atrial size. One of them had dynamic LV outflow tract obstruction associated with right ventricular dilatation. In the other, the LA dilatation was considered mild, with a mitral E:A ratio < 1.0 (0.79) and no evidence of LV hypertrophy.

### Comparison of echocardiographic features between cats with hyperthyroidism-associated HCMP to a contemporary sample of cats with primary HCM

The contemporaneous group of normotensive, euthyroid cats with primary HCM, similar to the hyperthyroidism-associated HCMP group (*n* = 94) with respect to age and BW, included 112 cats that met all required inclusion criteria (age = 12.0 years [10.0-14.0]; BW = 4.6 kg [4.0-5.0]; T4 = 24.0 nmol/L [18.2-33.3]; Figure 1).

**Figure 4:**
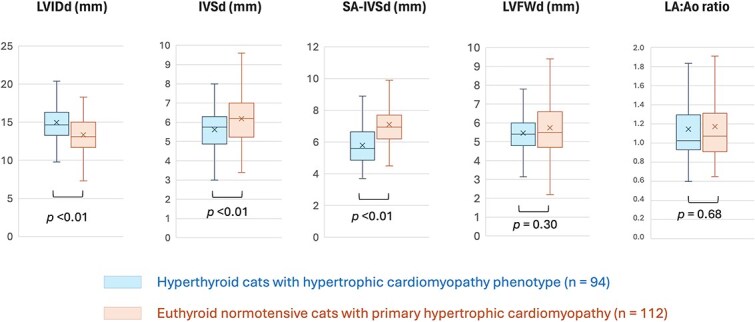
Box and whisker plots showing the comparison of LVIDd, IVSd, SA-IVSd, LVFWd, and LA:Ao ratio between cats with hyperthyroidism-associated hypertrophic cardiomyopathy phenotype and cats with primary hypertrophic cardiomyopathy. In each plot, the boxes represent the IQR (25th-75th percentiles), the horizontal line within the box represents the median, and the whiskers extend to the minimum and maximum values within 1.5 × IQR. Outliers beyond this range are not displayed. The “X” indicates the mean value. Abbreviations: IVSd = end-diastolic interventricular septal thickness; LA:Ao ratio = end-diastolic left atrium-to-aorta ratio; LVIDd = end-diastolic left ventricular internal diameter; LVFWd = end-diastolic left ventricular free wall thickness; SA-IVSd = end-diastolic subaortic interventricular septal thickness.

Regarding cardiac chamber measurements (Figure 4), the end-diastolic LV diameter was significantly higher in cats with hyperthyroidism-associated HCMP (14.7 mm [13.3-16.3]) than in cats with primary HCM (13.1 mm [11.7-15.0]; *P* < .01). This difference remained significant after normalization for both age and BW (*P* < .01). No significant difference was found in LA (*P* = .09) or LA:Ao ratio (*P* = .68) between cats with hyperthyroidism-associated HCMP (LA = 10.7 mm [9.2-13.0]; LA:Ao = 1.02 [0.93-1.29]; *n* = 94) and cats with primary HCM (LA = 11.3 mm [9.7-13.1]; LA:Ao = 1.07 [0.91-1.30]).

Regarding LV thicknesses (Figure 4), the end-diastolic IVS was significantly thicker (*P* < .01) in cats with primary HCM (6.2 mm [5.3-7.0]) than in those with hyperthyroidism-associated HCMP (5.8 mm [4.9-6.3]). This difference remained significantly different after normalizing for age (*P* < .01), but not after normalizing for BW (*P* = .15). In addition, subaortic IVS hypertrophy was significantly more frequent (*P* < .01) in primary HCM cats (95%) than in those with hyperthyroidism-associated HCMP (67%). The end-diastolic subaortic IVS thickness was also significantly higher in primary HCM cats (6.9 mm [6.2-7.7]) than in cats with hyperthyroidism-associated HCMP (5.6 mm [4.9-6.6]; *P* < .01). This difference remained significant after normalization for age (*P* < .01), but lost significance after normalization for BW (*P* = .27).

Regarding Doppler examination, no significant difference was found in the prevalence of dynamic LV outflow tract obstruction (25% vs 31%; *P* = .37) and in maximal aortic flow velocity (1.43 m/s [1.1-2.1] vs 1.45 m/s [1.1-2.4]; *P* = .86) between cats with hyperthyroidism-associated HCMP and primary HCM, respectively.

## Discussion

Hyperthyroidism, which is considered the most common endocrinopathy in middle-aged and older cats, is known to structurally and functionally affect multiple organs, including the cardiovascular system.^1–3,16–21^ In both human patients and cats, hyperthyroidism has been reported to induce HCMP, which can lead to CHF, but also may resolve over time after antithyroid treatment.^1–3,16–20^ The ACVIM consensus statement on cardiomyopathies in cats also suggests a possible association with RCMP.^4^ However, to the best of our knowledge, this association between hyperthyroidism and RCMP in cats remained unclear and the prevalence of RCMP in hyperthyroid cats had never been determined.^4,22^ Our study, conducted using a large sample of hyperthyroid cats, provides novel clinical and echocardiographic insights into hyperthyroidism-associated CMPs. Our results confirm that concurrent structural cardiac changes are common in hyperthyroid cats and indicate that, whereas HCMP is the predominant phenotype (80%), other forms, including RCMP and NSCMP, also are observed in a notable proportion of cases (20%).

We aimed to provide the most accurate description of cardiac abnormalities associated with naturally occurring hyperthyroidism in cats by applying strict inclusion criteria. These included precise and short duration of time between echocardiography, hyperthyroidism diagnosis, and initiation of antithyroid treatment, as well as the exclusion of cats with unknown dates of echocardiography or T4 measurement. Thus, the median intervals between echocardiography and hyperthyroidism diagnosis, and between the initiation of antithyroid treatment and echocardiography, were −3 days and 1 day, respectively.

Thyroid hormone excess can lead to myocardial hypertrophy in both humans and cats.^1–3,16–20^ The mechanisms responsible for such cardiac remodeling include both a direct effect of thyroid hormones on cardiomyocyte growth and interactions with other endocrine systems, such as the renin–angiotensin–aldosterone system and sympathetic nervous system.^19^ In our study, HCMP was observed in approximately two-thirds of hyperthyroid cats. Nevertheless, most of these cats (89%, 84/94) were classified as subclinical ACVIM stage B, with the majority (81%, 68/84) belonging to stage B1. Also, in HCMP cats, higher T4 concentrations were associated with more advanced ACVIM stages. Similarly, in the entire hyperthyroid sample, a positive correlation was found between T4 and end-diastolic LV diameter, LA, LA:Ao, and E wave velocity. These results support prior evidence that hyperthyroidism in cats adversely affects both their general health and the cardiovascular system, emphasizing the deleterious effects of this endocrinopathy.^2,23^

Most HCMP cats were hypertensive (79%), which might have contributed to a variable extent to myocardial hypertrophy.^12^ However, the prevalence and severity of systemic arterial hypertension were similar among cats with HCMP, RCMP, and even those without myocardial remodeling (No CMP group), suggesting that systemic hypertension in hyperthyroid cats is not the main trigger of myocardial hypertrophy nor the primary driver of a specific CMP.

In our study, hyperthyroid cats with HCMP were compared with a contemporaneous sample of cats with primary HCM. Even if T4 concentrations generally decrease with age,^24^ it is unlikely that some subclinical recently hyperthyroid cats have been included in the group of cats with primary HCM considering the distribution of the T4 concentrations in this group of 24.0 nmol/L (18.2-33.3). Interestingly, comparison with a contemporary sample of cats with primary HCM determined that hyperthyroid cats with HCMP are characterized by a larger end-diastolic LV diameter. This difference remained significant after normalization for both age and BW (*P* < .01). Notably, hyperthyroid cats with HCMP had lower BW (median = 3.7 kg) compared with cats diagnosed with primary HCM (median = 4.6 kg). Given that BW is positively correlated with most echocardiographic parameters in cats,^7,11^ this discrepancy would be expected to attenuate, rather than accentuate, the observed differences. Therefore, the persistence of significance despite the lower BW in the hyperthyroid group further supports the robustness and biological relevance of this finding. The latter may be explained, at least in part, by the combined direct and indirect growth-promoting effects of thyroid hormones on the myocardium associated with volume overload resulting from activation of the renin–angiotensin–aldosterone system and secondary aldosterone-mediated sodium and water retention.^19^ In addition, both the prevalence and severity of IVS hypertrophy were higher in cats with primary HCM than in hyperthyroid cats with HCMP. This finding is consistent with previously published data comparing pathological cardiac changes in cats with hyperthyroidism and those with primary HCM, showing that hyperthyroidism is associated with less pronounced gross cardiac hypertrophy and cardiomegaly than primary HCM.^25^ However, the difference in IVS hypertrophy severity assessed by both end-diastolic IVS and subaortic IVS thickness lost significance between groups after normalization for BW, indicating that BW acted as a potential confounding factor. We observed a discrepancy in that the prevalence of subaortic IVS hypertrophy was significantly higher in primary HCM cats (95%) compared with HCMP cats (67%), whereas the difference in absolute subaortic IVS thickness lost significance after normalization for BW. This discrepancy may be partly explained by the fact that we defined subaortic hypertrophy using the 95% prediction intervals of end-diastolic IVS thickness according to BW derived from M-mode measurements in the transventricular short-axis view,^11^ because, to the best of our knowledge, similar prediction intervals for 2D subaortic IVS thickness are not yet available in cats. Nevertheless, given the considerable overlap among groups for both LV end-diastolic diameter and IVS thickness (as illustrated in Figure 4), no reliable cut-off can be established to differentiate hyperthyroid-related changes from those associated with primary HCM. Nonsignificant results between HCMP and primary HCM cats (such as for dynamic LV outflow tract obstruction) likely reflect the clinical impression of similarity between groups for these specific variables, rather than a lack of power, and that increasing sample size would not be expected to identify clinically relevant differences. However, a nonsignificant difference cannot suggest, at the target population level, an absence of a real difference.^26^

In our study, the second most common CMP was RCMP, affecting 14% of hyperthyroid cats and 17% of those with CMP. Unlike cats with HCMP, most hyperthyroid cats with RCMP (80%) presented with CHF with or without arterial thromboembolism, and therefore were classified as ACVIM stage C. These cats had a significantly higher prevalence of pulmonary edema, pleural effusion, pericardial effusion, and intracardiac thrombus or arterial thromboembolism compared with the HCMP group. In addition, hyperthyroid cats with RCMP were characterized by significantly higher median T4 concentrations than those in the HCMP group (154 nmol/L vs 95 nmol/L, respectively). These findings support the hypothesis that the RCMP may reflect a more advanced or severe manifestation of hyperthyroidism in cats. As reported in both human patients and cats, and also in experimental studies,^18,25,27^ hyperthyroidism can induce severe cardiac histopathologic changes that include not only myocardial hypertrophy but also myocardial fibrosis and intramural arterial alterations, potentially leading to diastolic dysfunction. Such lesions could be involved in the pathophysiology of RCMP in hyperthyroid cats, a hypothesis that warrants confirmation through further histopathological studies.

Our study also provides valuable data regarding cardiac auscultation in a large referral sample of hyperthyroid cats. The overall prevalence of heart murmurs in our study sample of cats was high (84%), and also in the 3 most common echocardiographic groups (ie, HCMP [85%], RCMP [80%], and No CMP [87%]). Therefore, the presence of a heart murmur does not appear to be indicative of a specific CMP, although the median heart murmur grade was higher in the HCMP group (grade 4) compared with the RCMP (grade 2) and No CMP (grade 3) groups. Furthermore, 60% of RCMP cats (12/20) had a left apical systolic murmur, a prevalence similar to that reported in a previous study involving a large sample of cats diagnosed with primary RCM (63%).^8^

In addition, a gallop sound was detected only in cats with either HCMP (10%) or RCMP (25%). These results suggest, in accordance with previous studies and with the ACVIM consensus statement on cardiomyopathies in cats, that the presence of a gallop sound is more likely to be associated with a CMP and warrants further diagnostic investigations.^4,12,28,29^

Our study had some limitations. Firstly, a subset of the included hyperthyroid cats was referred by a specialized internal medicine department because of cardiac auscultation abnormalities detected at the time of hyperthyroidism diagnosis. This recruitment bias may have led to an overestimation of the prevalence of CMPs. For the same reason, the prevalence of heart murmur and gallop sounds also may have been overestimated. Other study limitations are inherent to the retrospective design. Thus, SBP measurements were available for the majority of hyperthyroid cats, but were missing in 23% of cases. Another important limitation concerns the assessment of disease severity. Correlations between the severity of hyperthyroidism and cardiac abnormalities were based solely on T4 concentrations, assuming it as the only marker of disease severity. However, this approach does not account for other potential indicators of severity or chronicity, such as the duration and intensity of clinical signs, BW, hepatic enzyme induction, or renal function status. This factor could have led to a simplified interpretation of the relationship between thyroid status and cardiac manifestations. Nevertheless, existing data support an association between T4 and the severity of hyperthyroidism, lending some validity to this approach.^2^ Another limitation lies in the 45-day cut-off, defined as the maximum allowable interval between echocardiographic examination and the diagnosis of hyperthyroidism, which was used as an inclusion criterion in our study. Previous studies have shown that cardiac abnormalities in hyperthyroid cats with HCM phenotype may regress partially or completely 3 months after achieving an euthyroid state, with a concomitant decrease in cardiac biomarkers (eg, N-terminal pro brain natriuretic peptide, cardiac troponin I).^3,30,31^ However, the exact timepoint at which regression of hyperthyroid-induced cardiac changes first becomes detectable by echocardiography is not clearly established in cats. For this reason, we considered it reasonable to use half of this 3-month interval (ie, 45 days) as a cut-off, to ensure that cats included in the study were evaluated close enough to the time of hyperthyroidism diagnosis or treatment initiation, while minimizing the risk of including cats in which regression of cardiac changes may already have occurred.

Finally, one of the main limitations of our study is that it focused exclusively on cardiac abnormalities at the time of hyperthyroidism diagnosis. Because it was based on a single evaluation, our study cannot establish a direct causal relationship between hyperthyroidism and the cardiac phenotypes observed. Future studies therefore should investigate the longitudinal course of hyperthyroid cats with cardiac structural changes to determine whether these phenotypes regress with antithyroid treatment (suggesting a causal link) or merely represent associations with the endocrinopathy. Additional studies also are needed to confirm our findings regarding the association between T4 and CMP as well as comparison of end-diastolic LV internal diameter and IVS hypertrophy between hyperthyroidism-associated HCMP cats and primary HCM cats.

## Conclusion

Our study provides a detailed clinical and echocardiographic characterization of cardiac abnormalities observed in a large sample of hyperthyroid cats at the time of diagnosis. Our findings confirm that HCMP is the predominant, but not sole, echocardiographic phenotype observed in hyperthyroid cats. The RCMP appears to be the second most frequent phenotype and may reflect a more severe form of the endocrinopathy.

## Off-label antimicrobial declaration

The authors declare no off-label use of antimicrobials.

## Institutional animal care and use committee or other approval declaration

The authors declare no institutional animal care and use committee or other approval was needed.

## Abbreviations

ACVIM: American College of Veterinary Internal Medicine
BW: body weight
CHF: congestive heart failure
CMP: cardiomyopathy phenotype
HCM: hypertrophic cardiomyopathy
HCMP: hypertrophic cardiomyopathy phenotype
IVS: interventricular septum
LA: left atrium
LA:Ao: left-atrium-to-aorta ratio
LV: left ventricle
LVFW: left ventricular free wall
No CMP group: group of hyperthyroid cats without myocardial abnormalities
NSCMP: nonspecific cardiomyopathy phenotype
RCMP: restrictive cardiomyopathy phenotype
SBP: systolic systemic arterial blood pressure
T4: total thyroxine
